# Perceptions and Attitudes of Mental Health Professionals toward the Mental Health Care Law in Saudi Arabia

**DOI:** 10.3390/healthcare11202784

**Published:** 2023-10-21

**Authors:** Ahmad H. Almadani, Eylaf S. Altheyab, Meshal A. Alkheraiji, Abdulaziz F. Alfraiji, Fatimah Albrekkan, AlRabab S. Alkhamis, Fay H. AlBuqami, Mohammed A. Aljaffer

**Affiliations:** 1 Department of Psychiatry, College of Medicine, King Saud University, Riyadh 11451, Saudi Arabia; falbrekkan@ksu.edu.sa (F.A.); maljaffer@ksu.edu.sa (M.A.A.); 2 Department of Psychiatry, King Saud University Medical City, King Saud University, Riyadh 11362, Saudi Arabia; ealtheyab@ksu.edu.sa (E.S.A.);; 3 SABIC Psychological Health Research and Applications Chair (SPHRAC), Department of Psychiatry, College of Medicine, King Saud University, Riyadh 12372, Saudi Arabia; 4 Department of Psychiatry, College of Medicine, Imam Abdulrahman Bin Faisal University, Dammam 31441, Saudi Arabia; askhamis@iau.edu.sa; 5 College of Medicine, King Saud University, Riyadh 11451, Saudi Arabia

**Keywords:** Mental Health Legislation Attitudes Scale, Saudi Mental Health Care Law, Saudi Arabia, Mental Health Act, mental health professionals

## Abstract

The Saudi Mental Health Care Law (SMHL) was established in 2014; however, no prior study has evaluated mental health professionals’ perceptions or attitudes toward it. This cross-sectional study examines these aspects and their associated factors among psychiatrists, psychologists, social workers, and psychiatric nurses in Saudi Arabia (SA). The data were collected from 432 participants using an online electronic survey consisting of four sections, including the Mental Health Legislation Attitudes Scale (MHLAS). Psychiatrists comprised most participants (46.06%), followed by psychologists (36.34%). Most participants were 40 years of age or younger (83.10%). Of the 432 participants, 226 (52.31%) were females. Overall, 28.70% were unaware of the existence of the SMHL. A total of 172 (66.67%) out of 258 participants agreed that the legislation operates well in ensuring treatment for persons who require involuntary admission. There was a statistically significant association between specialty and opinions of treatment efficacy and care benefits of the SMHL (*p* = 0.031 and *p* < 0.001, respectively). Official implementation of SMHL in participants’ facilities resulted in high MHLAS scores (*p* = 0.007). Reading or attending lectures and workshops related to the SMHL resulted in high MHLAS scores (*p* = 0.044 and *p* = 0.021, respectively). Negative opinions and uncertainty regarding the effect of SMHL on confidentiality were associated with low total MHLAS scores (*p* < 0.001). This study highlights the need to increase awareness of the SMHL among Saudi Arabian healthcare workers.

## 1. Introduction

The legal and ethical aspects of mental healthcare are crucial elements of day-to-day psychiatric practice [1]. Although laws applied in psychiatry and other medical fields are generally similar, treating patients with mental disorders not only involves unique clinical and ethical concerns but also holds unique legal implications [1,2]. Therefore, it is imperative to create well-recognized legislation and establish a holistic framework to streamline the process to be followed by mental healthcare workers during their clinical practice [3]. However, there has been much debate about the scope of mental health laws owing to the significant ethical and legal implications, especially regarding the administration of treatment without consent [4,5].

Unfortunately, besides developed countries, laws pertaining to the rights and management of patients with mental illnesses are lacking in many countries [3,6]. Establishing clear mental health legislation can be challenging for governments and legislating bodies [3]. This challenge arises from the necessity to craft comprehensive legislation to balance various stakeholders’ interests [3]. Such sought legislation also entails broad consultations of varied expertise to account for differing viewpoints to ensure that any laws enacted are comprehensive and thorough [3]. The earlier mentioned requirements can prove challenging to prioritize and uphold, particularly within politically dysfunctional areas amid threatened security or destabilized economies. 

Further, there is general agreement about the importance of the Mental Health Act (MHA) in developed countries [7]. In the UK, involuntary admission can be invoked if the patient’s health poses a risk to the safety of the patient and others [8]. Similarly, in Northern Ireland, involuntary admission is employed in the management of mental disorders that pose serious risks to the patient or others and is based on the necessity of treatment to prevent such risks [8]. Due to the constrictive and liberty-restrictive powers given to medical professionals in the MHA, there are countermeasures described in mental health laws to mitigate the misuse of involuntary admissions under the MHA [9]. These countermeasures include filing for an appeal to the appropriate appellate body within said country [9]. In the literature, the relevant procedures for filing for an appeal have been described in countries such as England, Wales, Scotland, Canada, and the United States of America [9].

One of the most extensive studies addressing involuntary admission of patients with mental illnesses has been conducted on a sample from 40 countries, all of which are part of the European Psychiatry Association. The authors found that significant differences concerning the use of the MHA among countries were mainly related to differences in the legal system rather than clinical diagnosis and treatment [7]. Furthermore, the authors highlighted significant differences in involuntary admission indicators across countries. For instance, although danger to oneself and danger to others were indicators of involuntary admission in 54% and 46% of the study population, respectively, a few countries, such as Italy and Spain, have omitted the “danger to self or others” criterion for involuntary admissions [7]. Likewise, in Sweden, a patient can be involuntarily admitted if the decision is deemed to be in the patient’s best interest, irrespective of the risk of danger [6].

Georgieva et al. [6] recently conducted a study examining significant inter-country differences in mental health laws. Using a brief nine-item Mental Health Legislation Attitudes Scale (MHLAS), the study explored opinions about the MHA among stakeholders in 11 countries. Participants included mental health workers (e.g., doctors and nurses), non-mental health workers (e.g., police officers), and patients’ families [6,10]. Doctors and nurses were generally more satisfied with the current mental health laws than were the police and family members of patients [6]. 

Saudi Arabia (SA) had no unifying governmental mental health statute in effect prior to 2014 [11,12,13,14]. However, efforts were made to mitigate such gaps in the mental healthcare system; for example, incident-driven human rights committees were established to mitigate human rights complaints [13,14]. These committees also provided the government with suggestions regarding legislation, mental health policies, quality assessments, monitoring, and service planning [13,14]. The MHA is important since it provides a clear, unified framework under the supervision of the government [11,14]. 

A comprehensive hospital-based mental health system has been developed in Saudi Arabia during the last three decades. Subsequently, the Saudi Mental Health Care Law (SMHL) passed legislation in 2014 [13], which includes several international human rights standards promoted by the World Health Organization (WHO) [15]. It is worth mentioning that the WHO has proposed the “Mental Health Action Plan 2013–2020” to reduce the morbidity, mortality, and disability of people with mental illnesses by promoting mental well-being and human rights, and preventing mental diseases [16]. The plan consists of six principles: universal health care, human rights, evidence-based practice, life-course approach, multisectoral approach, and empowerment of persons with mental disorders and psychosocial disabilities. This plan has been proposed to all WHO member states, international partners like the World Bank and the United Nations development agencies, and national partners like regional development banks [16]. The plan also calls for implementing fundamental principles in mental health law to promote human rights and encourage the development of accessible social and health services. The Mental Health Action Plan and the WHO Special Initiative for Mental Health call for mental health issues to be included in priority health programs [16,17]. 

Further, in 2016, Queensland’s MHA underwent significant reforms, focusing on the legal and human rights of people with mental illnesses [18,19]. The act provides a framework for dealing with mental health issues while maintaining human and legal rights [19]. This act detailed and highlighted the importance of accommodating people with mental illnesses by supporting their decisions and providing treatment in the least restrictive way possible. Family members or caregivers are crucial in the individual’s care and treatment decisions. The act also considers cultural and linguistic backgrounds, highlighting the importance of the assistance of an interpreter. It also emphasizes the best interest of minors, meeting their specific needs and providing treatment separately from adults if possible. Recovery-oriented services are also emphasized, along with reducing the stigma associated with mental illnesses [19]. 

The Saudi Mental Health Care Law (SMHL) is a statute currently governing the mental healthcare system in Saudi Arabia. The SMHL, specifically in Article 9, states the various rights of psychiatric patients, such as the right to treatment in a safe and clean environment, prior information about the treatment plan, assurance that they cannot be treated without their consent (or the consent of their guardian if they are incapable of determining their need for treatment), and information about the involuntary admission, the process of revoking the decision, and, if detained, the right to be held by the least restrictive means. The law also states that patients’ personal information should be kept confidential and not disclosed unless requested by the general or local mental health supervisory board—at the request of judicial or investigation authorities—and the reasons for obtaining such confidential information are stated. Furthermore, the patients or their guardians have the right to file any complaints against anyone in a mental health treatment facility, if justified, without affecting their quality of care [13]. 

The SMHL defines a mental disorder (Article 1) as “a disturbance in an individual’s thought, mood, awareness, memory or other intellectual faculties, in whole or in part” [20] (p. 1). The SMHL also clearly defines the indications for the admission of patients diagnosed with mental illnesses [13,20]. According to Article 13 of the SMHL, the criteria for involuntary admission are as follows: the patient must demonstrate signs of severe mental illness and be at risk of harming self or others, and the admission must be deemed necessary to improve and control any deterioration in the patient’s condition [20]. If both these conditions are met, two psychiatrists must sign a written statement stating that the criteria of Article 13 have been met [20]. Moreover, Article 17 of the SMHL also allows the involuntary treatment of patients with psychiatric illnesses if two psychiatrists approve and justify the involuntary treatment [20]. 

Evaluating mental healthcare providers’ perceptions of this law is imperative for providing high-quality clinical care to their patients while strictly following government legislation [2]. Despite the SMHL being in place since 2014 [21], to the best of our knowledge, no prior study has evaluated the perspectives of mental health workers on this law. Therefore, this study aims to explore the perceptions and attitudes of mental healthcare workers toward the SMHL, as well as the factors influencing their perceptions and attitudes. 

## 2. Materials and Methods

### 2.1. Study Design, Participants, and Setting

This cross-sectional study was conducted at the national level in Saudi Arabia, with the study population consisting of mental health professionals. The inclusion criteria included psychiatrists, psychologists, nurses, and social workers in Saudi Arabia, including those practicing and training in these disciplines. Based on the available data from the 2021 Statistical Yearbook of the SA Ministry of Health [22], the estimated targeted population was 202,380 (psychiatrists: 1350; psychologists: 1036; social workers: 3199; and nurses: 196,795). Convenience sampling was used to recruit participants. Using a sample size calculator (Raosoft, http://www.raosoft.com/samplesize.html, accessed on 3 October 2022) with a 95% confidence interval (CI) and a 5% margin of error, we estimated the required sample size to be 384 participants.

### 2.2. Survey Scale

The study used an electronic survey consisting of four sections. The first section included questions on demographic and practical information. The second section comprised questions that assessed SMHL awareness. If the participant was unaware of the existence of the SMHL, the survey was programmed to end. However, if the participant was aware of the law, the survey was programmed to proceed to section three, which contained questions on the MHLAS [6,10]. The fourth section contained questions on SMHL implementation and confidentiality. 

The MHLAS consists of nine elements [6,10], evaluating the following:

- Question (Q)1 (treatment efficacy): The legislation operates well in ensuring treatment for persons who require involuntary admission.
- Q2 (admission criteria): The clinical assessment required to meet the criteria for involuntary admission works well under the legislation.
- Q3 (care benefits): Individuals admitted without their consent generally benefit from the care received.
- Q4 (consent to treatment): The law supports individuals’ rights to refuse or consent to treatment, where possible.
- Q5 (detention review): The law supports the fair and independent review of an individual’s detention.
- Q6 (implementation of the law): The law is difficult to implement in clinical practice.
- Q7 (information about the law): Information about the law is not readily available.
- Q8 (transfer to hospital): The transfer to the inpatient ward works well under the law.
- Q9 (reciprocity principle): Individuals admitted without their consent receive both the most effective and the least restrictive care available under the circumstance.

Each question on the MHLAS was rated on a five-point Likert scale, ranging from strongly disagree to strongly agree. The higher the score, the more the answers reflected a positive attitude toward the national mental health law. However, Q6 and Q7 were reverse-scored. The MHLAS has been developed by an interdisciplinary team of professionals specializing in ethics, mental health law, psychiatry, general nursing, mental health nursing, and service users. The diversity of the team establishing the scale offers multidimensional viewpoints to it and, therefore, facilitates the adequate understanding and capturing of the opinions regarding the law. Moreover, the MHLAS has been used in many countries; thus, it can pave the way for comparison of the results of this study with those of other countries across the globe [6,10]. As such, the MHLAS was adopted in this study, and permission to use it was obtained from its authors [6,10].

### 2.3. Procedure and Data Collection

As elaborated in the previous section, an online survey consisting of four segments was administered using Momentive’s Survey Monkey (https://www.surveymonkey.com, accessed on 3 October 2022). The web link to the survey was sent between the beginning of January and the end of February 2023 to the participants by the research team and data collectors through their professional networks and social media platforms, such as WhatsApp through direct communication or via WhatsApp cluster groups containing those practicing and training in each region in Saudi Arabia. The nature and purpose of the study, the principal investigator’s contact information, and an explanation of the confidentiality and data anonymity policies were provided. Consent to participate was obtained through a click on an informed consent link. After reading the informed consent statement, the participants clicked “Next” to access the study’s survey, which took approximately 5 min to complete. The study received ethical approval from the Institutional Review Board at the College of Medicine at King Saud University, Riyadh (research project number: E-22-7386), on 15 December 2022, and it conformed to the principles of the Declaration of Helsinki.

### 2.4. Statistical Analysis

All statistical analyses were performed using SPSS version 28 (IBM Corp., Armonk, NY, USA). Quantitative parametric data were presented as means and standard deviations, whereas quantitative non-parametric data were presented as medians and interquartile ranges and analyzed using the Kruskal–Wallis test. Categorical variables were presented as frequencies and percentages (%) and analyzed using a chi-square test. Mean indices of each domain of the MHLAS were calculated to assess this study’s latent variable (i.e., the attitude toward the SMHL). Ordinal logistic regression was performed to determine the factors associated with the different MHLAS domains. Linear regression was performed to ascertain factors associated with the total MHLAS score. Statistical significance was defined as a two-tailed test with *p* < 0.05. 

## 3. Results

The demographics and knowledge of the SMHL are presented in Table 1 for all 432 participants, among whom there were 57 nurses, 199 psychiatrists, 157 psychologists, and 19 social workers. A comparison among specialty groups revealed statistically significant (*p* < 0.05) differences in age, sex distribution, nationality, current region of practice, level and type of service facility in the hospital, years of experience, current role at the time of the study, and awareness of the existence of the SMHL. 

**Figure 1:**
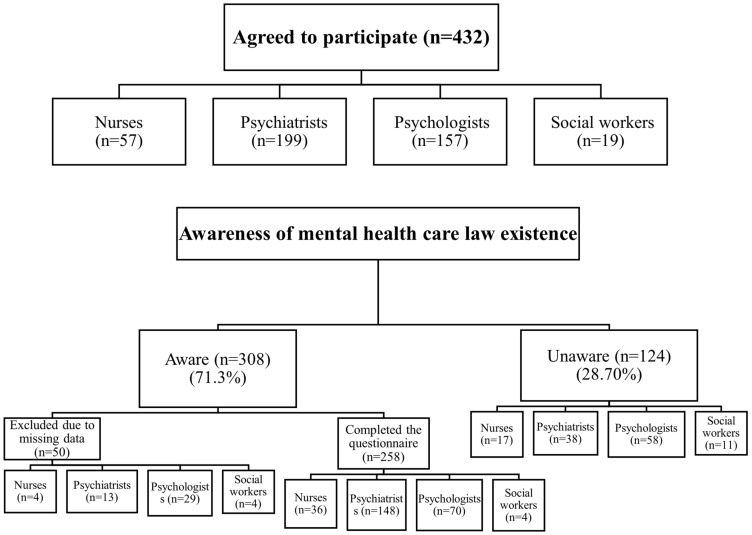
Flowchart of participant recruitment.

Of the 432 people who agreed to participate in this study, 308 (71.3%) were aware of the SMHL, and 124 (28.70%) were unaware. Among those who were aware of the legislation, 50 were excluded from further analysis of the MHLAS scores because of incomplete responses to the survey in section three (MHLAS) and beyond, representing (11.57%) of the total sample (432) and (16.23%) of those aware of the legislation (Figure 1). Psychiatrists showed the highest percentage of awareness of the SMHL at 161 (80.90%), followed by nurses at 40 (70.18%), psychologists at 99 (63.06%), and social workers at 8 (42.11%).

**Figure 2:**
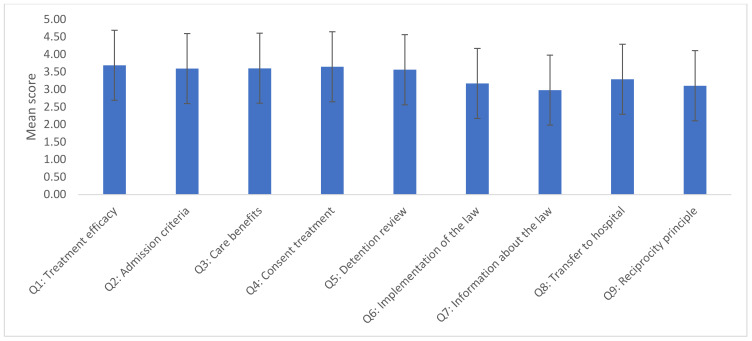
Participants’ opinions toward the Saudi Mental Health Care Law across different domains.

Table 2 presents the opinions of the participants regarding the SMHL, expressed as percentages, total average agreement score, and standard deviation. The higher the score, the more the item was agreed upon. The highest level of agreement was observed for Q1, “The legislation operates well in ensuring treatment for persons who require involuntary admission”, with an average score of (3.69 ±1.03), and a majority of agreeing participants comprising 66.67%. The lowest level of agreement was observed for Q7, “Information about the law is not readily available”, with an average score of 2.98 ± 1.12 (Figure 2).

As summarized in Table 3, there was a statistically significant influence of specialty and opinions on treatment efficacy and care benefits of the SMHL (*p* = 0.031 and *p* < 0.001, respectively); psychiatrists scored significantly higher in the treatment efficacy domain than did the psychologists (*p* = 0.004), and psychiatrists scored significantly higher in the care benefits domain than did nurses and psychologists (*p* = 0.022 and *p* < 0.001, respectively).

As shown in Table 4, ordinal logistic regression models were used to study the factors associated with the agreement level. The responses to Q1 regarding treatment efficacy suggested that participants who believed that the healthcare law negatively affected patients’ confidentiality and those who were unsure were less likely to agree on this domain than those who believed otherwise (odds ratios (*OR*s) = 0.2 and 0.37, respectively). Participants who stated that the law was neither implemented nor followed in their facilities were less likely to agree with this domain than those with uncertain opinions (*OR* = 0.24). In addition, participants who had previously attended lectures and workshops on the SMHL were more likely to agree in this domain than were those who had not (*OR* = 1.88).

The responses to Q2 about admission criteria indicated that practicing participants in the northern region were more likely to agree with this domain than were those in the central region (*OR* = 3.3). Participants who believed that the healthcare law negatively affected patients’ confidentiality and those who were unsure were less likely to agree in this domain than those who believed otherwise (*OR*s = 0.44 and 0.42, respectively). Participants who stated that the law was neither implemented nor followed in their facilities were less likely to agree in this domain than were those with uncertain opinions (*OR* = 0.33). The responses to Q3 on care benefits indicated that psychiatrists were more likely to agree than nurses (*OR* = 2.47).

The responses to Q4 about consented treatment suggested that participants who believed that the healthcare law negatively affected patients’ confidentiality and those who were unsure were less likely to agree on this domain than those who believed otherwise (*OR*s = 0.35 and 0.47, respectively). Participants who stated that the law was neither implemented nor followed in their facilities were less likely to agree with this domain than those with uncertain opinions (*OR* = 0.32). In addition, participants who intended to attend lectures and workshops were less likely to agree with this domain than those who did not (*OR* = 0.52).

The responses to Q5 about detention review indicated that practicing participants in the northern region were more likely to agree with this domain than were those in the central region (*OR* = 5.33). Participants who believed that the healthcare law negatively affected patients’ confidentiality and those who were unsure were less likely to agree in this domain than those who believed otherwise (*OR* = 0.35 and 0.36, respectively). Participants who stated that the law was neither implemented nor followed in their facilities were less likely to agree in this domain than were those with uncertain opinions (*OR* = 0.27).

The responses to Q6 about the law’s implementation suggested that participants who believed that the healthcare law negatively affected patients’ confidentiality and those who were unsure were less likely to agree in this domain than those who believed otherwise (*OR* = 0.24 and 0.42, respectively). The responses to Q7 (information about the law) indicated that participants with an uncertain opinion were less likely to agree than those who believed that the law had no adverse effect on confidentiality (*OR* = 0.49).

The responses to Q8 regarding transfer to the hospital suggested that practicing participants in the northern regions were more likely to agree than were those in the central regions (*OR* = 3.82). Participants who believed that the healthcare law negatively affected patients’ confidentiality were less likely to agree on this domain than those who believed otherwise (*OR* = 0.43). The responses to Q9 on the reciprocity principle indicated that Saudi participants were less likely to agree than non-Saudi participants (*OR* = 0.33). Participants who stated that the law was officially implemented in their facilities were more likely to agree in this domain than were those with uncertain opinions (*OR* = 2.09).

According to the results of simple regression analysis, participants who believed that the healthcare law negatively affected patients’ confidentiality and those who were not sure had significantly lower scores than those who believed otherwise (coefficient = −4.37, 95% CI [−6.32, −2.43], *p* < 0.001, and coefficient = −3, 95% CI [−4.36, −1.64], *p* < 0.001, respectively). Participants who stated that the law was officially implemented in their facilities had a significantly higher score than those with an uncertain answer (coefficient = 2.25, 95% CI [0.62, 3.88], *p* = 0.007). Compared with participants who had never read about the law nor attended any lecture or workshop about it, those who had done either of these activities had significantly higher scores (coefficient = 1.99, 95% CI [0.05, 3.93], *p* = 0.044, and coefficient = 1.81, 95% CI [0.27, 3.35], *p* = 0.021, respectively).

Based on the results of multiple regression (Table 5), opinion on confidentiality was significantly associated with the total MHLAS score as participants who believed that the healthcare law negatively affected patients’ confidentiality and those who were not sure scored significantly lower than had those who believed the opposite (coefficient = −4.26, 95% CI [−6.28, −2.24], *p* < 0.001, and coefficient = −2.67, 95% CI [−4.1, −1.24], *p* < 0.001, respectively). Participants who stated that the law was neither implemented nor followed in their facilities had a significantly lower score than those with an uncertain opinion (coefficient = −3.71, 95% CI [−6.35, −1.06], *p* = 0.006).

## 4. Discussion

This study is the first to explore mental health professionals’ perceptions and attitudes toward the SMHL. The limited availability of literature on the subject presents an opportunity to enhance our understanding by exploring the various domains and health workers’ perceptions, with the potential for future improvements in our current practices to protect the rights of people receiving mental health services. 

Our results revealed that 28.70% of mental health professionals were unaware of the SMHL, which could be attributed to multiple factors. The first factor is the relatively recent passage of the law, which is still undergoing multiple revisions [13,20] compared to other countries [8,9]. For example, the United States of America first signed an act to facilitate the treatment of mentally ill patients in 1963, while Italy implemented a law in 1978 to regulate the voluntary and mandatory treatment of the mentally ill [8]. Moreover, England and Wales introduced the MHA in 1959 [9]. Second, despite the availability of the SMHL on the Ministry of Health’s website [20], the majority of participants in our study agreed with Q7, “Information about the law is not readily available”, which highlights the need for a different means of raising the awareness and knowledge about the legislation in such population. Moreover, there is a probable lack of supervisory auditing on mental health facilities. Last, mandatory educational workshops or lectures concerning the SMHL are lacking for those practicing or in training. Altogether, these factors might have contributed to the lack of SMHL awareness among the participants. Further, while psychiatrists had the highest awareness rate among all the participants, social workers had the lowest awareness rate. Our findings concerning social workers differ from those of another study conducted in the Philippines, which aimed to assess the awareness of mental health laws among registered social workers [23]. Their findings revealed that social workers, as a group, were highly aware of the mental health law [23]. This difference can be attributed to the experience of using the law in practice, which could influence awareness and knowledge of the legislation. Thus, it can be inferred that the actual involvement of social workers in the development of the SMHL appears to remain poor, which, subsequently, could explain their limited awareness. Another explanation for our findings concerning social workers could be related to the limited number of participants in our sample, making it challenging to conclusively identify significant results within this group or generalize the findings to other social workers in the country.

Concerning the official implementation of the SMHL, approximately half of the participants in our study reported that the SMHL has not been officially implemented in their facilities. This finding is worrisome, as facilities ought to follow the policies and procedures established in the legislation [21]. Given the low official implementation of the SMHL, it is important to identify facilities where the policies and procedures of the SMHL are not being followed and explore the reasons behind this deviation from expected practice. In Saudi Arabia, the “General Supervisory Board for Mental Health Care”, as stated in Article 4 of the SMHL, is responsible for overseeing the law’s implementation, ensuring compliance and monitoring of all facilities providing mental healthcare, and examining and verifying the records and reports to take the necessary action for rectifying any violations [21]. As per Article 4, this board is also responsible for proposing ideas for improving the SMHL and setting up local supervisory boards as needed [21]. 

Among the MHLAS domains, the highest and lowest levels of approval were observed in the treatment efficacy domain (Q1) and the information about the law domain (Q7), respectively. This finding contrasts with a previous study conducted by Georgieva et al. [6], where they examined the variations in mental health law regulations in 11 countries and reported the highest rate of agreement for the care benefits domain (Q3), while the lowest rate was observed for the implementation of the law domain (Q6). As the authors of that study explained, most countries struggle to organize mental health services and follow legal requirements [6]. Nevertheless, in our study, psychiatrists had significantly higher levels of agreement than other stakeholders (psychologists, nurses, and social workers) in the treatment efficacy (Q1) and care benefits (Q3) domains. This notably higher level of agreement among psychiatrists in our study may be owing to their mandatory engagement with the legislation in facilities that have officially implemented the law. 

Our study showed no statistically significant results when assessing the overall MHLAS scores across stakeholder groups (nurses, psychiatrists, psychologists, and social workers), which may reflect a balanced perception among the stakeholders. Georgieva et al. [6] showed that doctors and nurses were more likely to agree with the overall usefulness of mental health laws in their countries. Another study comparing the attitudes of mental health professionals and laypeople regarding involuntary admission and treatment in England and Germany showed that social workers in Germany and psychologists in England were less likely to support involuntary placement than doctors and nurses [24]. These results might be because social workers and psychologists were not involved in the detention process in their respective countries [24]. Another explanation we hypothesize could be that psychologists and social workers are more likely to adopt different perspectives and attitudes to mental distress than the biomedical model. For instance, the Power Threat Meaning Framework is an alternative approach to understanding and addressing psychological distress [25], which may lead to less support for involuntary admission.

Interestingly, in our study, professionals in the northern Saudi regions were more likely to agree with the admission criteria, detention reviews, and hospital transfers than were professionals in other regions. First, regarding admission criteria, professionals practicing in the northern regions believed that the clinical assessment and criteria for involuntary placement under the SMHL were adequate. Our findings align with the research outcomes of some countries included in Georgieva et al.’s [6] study, in which all countries except Slovenia and Ireland had a positive attitude toward the criteria for involuntary admission. The Irish Mental Health Law explicitly excludes patients with drug addiction or personality disorders from involuntary placement even if they are considered at risk to themselves or others, which may explain the disagreement with this aspect of the law [6]. Second, regarding detention reviews, professionals in the northern regions were more likely to agree that mental health legislation provided an independent and fair review process for patient detention than professionals from other regions. This result contrasts with the findings of a previous study conducted in Ireland on the opinions of stakeholders (including psychiatrists, nurses, general practitioners, and first-degree relatives of patients detained under the MHA) regarding involuntary admission [10]. The Irish study revealed greater dissatisfaction among family members concerning the fairness of detention review than among other stakeholders, implying the importance of involving family members in the care plan throughout the patient’s admission [10]. Finally, regarding transfer to hospitals, professionals in the northern regions also agreed with how the legislation transferred people to inpatient units. Contrary to our findings, Georgieva et al. [10] noted general dissatisfaction with the process and time required to transfer patients. The difference in the perception of these domains in the northern region in our study compared to that in Georgieva et al.’s [10] study could be due to unclear SMHL regulations regarding transfer to hospitals. This could also be explained by the small sample size from the northern Saudi Arabian region in our study.

Additionally, the results of our study demonstrated that compared to non-Saudi, the Saudi professionals, who also represented the majority of our study population, agreed less with the reciprocity principle, thus reflecting Saudi professionals’ disagreement with involuntarily admitted patients receiving the least restrictive and most effective care available. Our findings regarding the reciprocity principle are inconsistent with Georgieva et al.’s [10] results, which revealed that most stakeholders agreed that patients admitted involuntarily received the least restrictive and most effective care available. However, it is essential to note that in our study, many professionals stated that the SMHL was not officially implemented in their facilities, which might have impacted their perceived experience, affecting their belief in the legislation’s efficacy in providing the least restrictive measures. We found that the official implementation of the SMHL by facilities significantly increased the overall agreement, as reflected in the total MHLAS scores.

Regarding confidentiality, our results showed that practitioners who believed that the SMHL could negatively affect patients’ confidentiality disagreed in most domains. These results reflect the significance of maintaining patients’ privacy and ensuring they are well protected by the law. Nonetheless, confidentiality has been shown to be a crucial element in mental healthcare [26]. In our study, professionals who stated that the law was neither implemented nor followed in their facilities were less likely to agree with most domains and had significantly lower scores than those who followed the law. These findings are consistent with the results of Georgieva et al. [6], who revealed that the less experience participants had with involuntary admissions, the less likely they were to agree with most domains. 

Furthermore, a lack of exposure to mental health laws makes it easier to criticize such laws [23]. In our study, those who had never read the SMHL or attended lectures or workshops about the law had significantly lower scores than those who had. This finding highlights the need to provide such educational activities for healthcare workers. The need to offer such activities aligns with Fiorillo et al.’s [27] recommendation that regular training courses should be provided to professionals involved in the detention process.

We recommend that future studies should examine elements that have not been addressed in the current paper, such as the obstacles faced by the institutions in implementing the SMHL, the attitudes and awareness of other stakeholders, such as emergency physicians, and those who do not necessarily work in the mental health field, such as law enforcement personnel. Second, we recommend exploring stakeholders’ opinions on how to improve their satisfaction with the law. Third, we recommend including an Arabic questionnaire aiming to achieve a better representation of nurses and social workers in the country. Fourth, we recommend future studies to assess the perspective of patients receiving compulsory care and those directly or indirectly impacted by the law, such as patients’ families. Last, future studies should explore strategies to increase SMHL awareness, maintain high awareness, and ensure periodic monitoring. One approach is to conduct educational activities concerning the SMHL to target all mental health workers, highlighting the role of the legislation and emphasizing the confidentiality it ensures. Examples of scholarly activities include webinars, courses, and workshops. In contrast, the legislation in England and Wales, Section 12 of the MHA, mandates accreditation of induction and a periodic refresher course granted by the Secretary of State and delegated to local Section 12 panels [28,29,30]. Similarly, this can be applied to newly appointed mental health professionals in Saudi Arabia to complete an online program within a defined period of joining psychiatric services or not allowing them to commence work until they obtain accredited SMHL training. Local administrators should ensure staff are trained in the Act and require them to undergo refresher courses periodically, for which on-demand didactic webinars could be utilized. These measures should be implemented nationally to ensure the best outcomes and the highest awareness. Implementing these recommendations could provide more accessible information about the SMHL and serve as a tool for monitoring and auditing the law’s implementation. Nonetheless, including other relevant groups and agencies, such as the police, would be of significant value.

## 5. Conclusions

This study contributes to the literature by highlighting mental healthcare professionals’ current awareness and attitude toward the SMHL, providing valuable insights into factors influencing them, and identifying areas of dissatisfaction. This study emphasizes the considerably low official implementation of the SMHL and indicates that 28.70% of mental healthcare professionals need to be made aware of its existence. Nonetheless, the results show that significant difficulties still exist in the implementation process of the SMHL. Given the results of our study, it is crucial to ensure the official implementation of the legislation in all healthcare facilities in Saudi Arabia and to increase awareness among mental health professionals.

## 6. Strengths and Limitations

Our study has several strengths. One of them is that it is the first of its kind conducted at the national level in Saudi Arabia, allowing us to understand the perspectives of healthcare professionals who practice in various regions of the country and make more appropriate suggestions concerning the implementation of the SMHL. It also includes the use of a scale that has been utilized in previous studies [6,10], with one of them being conducted in 11 countries among people with varying cultural differences and backgrounds [6]. The use of this scale in our study supports our goal of offering a relative point of comparison with international statistics, which facilitates addressing the perceived shortcomings of the current SMHL. Another strength is that the sample size in our study was sufficient to provide an initial overview of the participants’ knowledge, perceptions, and attitudes toward the SMHL.

Our study also has certain limitations, including the need for more validation studies on the survey instrument, that is, the MHLAS [6,10], even though a multidisciplinary team developed it, and it has been used in more than one study [10]. Moreover, the MHLAS focuses on attitudes; thus, the current study did not assess knowledge about the details of admission criteria. Another area for improvement is related to the low enrolment rate of the nurses and social workers in the study compared to that of the other groups of participants. The language of the survey can partially explain this inadequate representation of the aforementioned groups, especially the social workers’ group, as the vast majority of social workers in the country speak Arabic and need more English language skills. Third, the sample population included only mental health professionals. However, other specialists, such as emergency physicians, can initiate some aspects of the SMHL, such as involuntary detention [21]. We opted to limit our study to mental health professionals for several reasons, including access to the study participants. Finally, both the southern and northern regions’ sample sizes were small compared to those from other areas, which could be due to, in part, the lack of implementation and knowledge of the SMHL in those parts of the country.

## Figures and Tables

**Table 1:** Participants’ demographics and knowledge of the Mental Health Care Law.

	**Specialty**				**Total**	***p*** **-Value**			
	**Nursing** **(n = 57)**	**Psychiatry** **(n = 199)**	**Psychology** **(n = 157)**	**Social Work** **(n = 19)**					
Age (years)									
<30	11 (19.30%)	79 (39.70%)	89 (56.69%)	3 (15.79%)	182	<0.001			
30–40	36 (63.16%)	83 (41.71%)	48 (30.57%)	10 (52.63%)	177				
41–50	6 (10.52%)	19 (9.55%)	14 (8.92%)	4 (21.05%)	43				
>50	4 (7.02%)	18 (9.04%)	6 (3.82%)	2 (10.53%)	30				
Sex									
Male	39 (68.42%)	129 (64.82%)	30 (19.11%)	8 (42.11%)	206	<0.001			
Female	18 (31.58%)	70 (35.18%)	127 (80.89%)	11 (57.89%)	226				
Nationality									
Non-Saudi	18 (31.58%)	25 (12.56%)	4 (2.55%)	2 (10.53%)	49	<0.001			
Saudi	39 (68.42%)	174 (87.44%)	153 (97.45%)	17 (89.47%)	383				
Current region of practice									
Central	26 (45.61%)	83 (41.71%)	110 (70.06%)	10 (52.63%)	229	<0.001			
Eastern	12 (21.05%)	73 (36.68%)	21 (13.38%)	3 (15.80%)	109				
Northern	4 (7.02%)	6 (3.01%)	8 (5.10%)	1 (5.26%)	19				
Southern	8 (14.04%)	10 (5.03%)	3 (1.91%)	1 (5.26%)	22				
Western	7 (12.28%)	27 (13.57%)	15 (9.55%)	4 (21.05%)	53				
Level of the hospital facility									
Primary care	18 (31.58%)	20 (10.05%)	68 (43.31%)	7 (36.84%)	113	<0.001			
Secondary hospital	11 (19.30%)	54 (27.14%)	46 (29.30%)	4 (21.05%)	115				
Tertiary hospital	28 (49.12%)	125 (62.81%)	43 (27.39%)	8 (42.11%)	204				
Facility service type									
Governmental sector	53 (92.98%)	189 (94.97%)	77 (49.04%)	14 (73.68%)	333	<0.001			
Private sector	4 (7.02%)	10 (5.03%)	80 (50.96%)	5 (26.32%)	99				
Years of experience in the field									
0–2 years	14 (24.56%)	44 (22.11%)	70 (44.59%)	4 (21.05%)	132	<0.001			
>2–5 years	4 (7.02%)	71 (35.68%)	45 (28.66%)	2 (10.53%)	122				
>5–10 years	14 (24.56%)	34 (17.08%)	23 (14.65%)	5 (26.32%)	76				
>10 years	25 (43.86%)	50 (25.13%)	19 (12.10%)	8 (42.10%)	102				
Current role of nurses, psychologists, and social workers									
In-training/internship	3 (5.26%)	---	39 (24.84%)	3 (15.79%)	45	0.005			
Practicing	54 (94.74%)		118 (75.16%)	16 (84.21%)	188				
Professional rank of psychiatrists									
Consultant	---	44 (22.11%)	---	---	---	---			
Senior registrar		31 (15.58%)							
Registrar		15 (7.54%)							
Resident		109 (54.77%)							
Awareness of the existence of the Mental Health Care Law									
Unaware	17 (29.82%)	38 (19.10%)	58 (36.94%)	11 (57.89%)	124	<0.001			
Aware	40 (70.18%)	161 (80.90%)	99 (63.06%)	8 (42.11%)	308				
**For those who said that they are aware of the law:**									
	**Nursing**			**Psychiatry**		**Psychology**	**Social work**	**Total**	***p*-value**
Read the law	(n = 40)			(n = 158)		(n = 97)	(n = 8)	303	
No	9 (22.50%)	16 (10.13%)	18 (18.56%)	2 (25.00%)	45				0.306
No, but planning to	12 (30.00%)	55 (34.81%)	27 (27.84%)	3 (37.50%)	97				
Yes	19 (47.50%)	87 (55.06%)	52 (53.61%)	3 (37.50%)	161				
Attends lectures and workshops	(n = 40)			(n = 158)		(n = 97)	(n = 8)	303	
No	14 (35.00%)	41 (25.95%)	31 (31.96%)	3 (37.50%)	89				0.709
No, but planning to	9 (22.50%)	44 (27.85%)	26 (26.80%)	3 (37.50%)	82				
Yes	17 (42.50%)	73 (46.20%)	40 (41.24%)	2 (35.00%)	132				
Opinion about confidentiality	(n = 36)			(n = 148)		(n = 70)	(n = 4)	258	
The law has no negative effect on patients’ confidentiality.	18 (50.00%)	91 (61.49%)	30 (42.86%)	2 (50.00%)	141				0.079
The law has a negative effect on patients’ confidentiality.	8 (22.22%)	13 (8.78%)	11 (15.71%)	0 (0.00%)	32				
I’m not sure.	10 (27.78%)	44 (29.73%)	29 (41.43%)	2 (50.00%)	85				
Facility implementing the mental health law	(n = 36)			(n = 148)		(n = 70)	(n = 4)	258	
Not sure	4 (11.11%)	30 (20.27%)	19 (27.14%)	2 (50.00%)	55				0.184
Neither implemented nor followed	2 (5.56%)	18 (12.16%)	2 (2.86%)	0 (0.00%)	22				
Followed but not implemented officially	7 (19.44%)	29 (19.59%)	15 (21.43%)	0 (0.00%)	51				
Officially implemented	23 (63.89%)	71 (47.97%)	34 (48.57%)	2 (50.00%)	130				

Note: Data are presented as frequency (%); statistical significance was set at *p*-value < 0.05.

**Table 2:** Participants’ opinions about the Saudi Mental Health Care Law.

	Strongly Disagree	Disagree	Neither Agree nor Disagree	Agree	Strongly Agree	Total	Score (Mean ± SD)
Q1: The legislation operates well in ensuring treatment for persons who require involuntary admission.	16 (6.20%)	11 (4.26%)	59 (22.87%)	122 (47.29%)	50 (19.38%)	258	3.69 ± 1.03
Q2: The clinical assessment to meet the criteria for involuntary admission works well under the legislation.	16 (6.20%)	14 (5.43%)	60 (23.26%)	135 (52.33%)	33 (12.79%)	258	3.60 ± 0.99
Q3: Individuals admitted without their consent generally benefit from the care received.	11 (4.26%)	30 (11.63%)	61 (23.64%)	103 (39.92%)	53 (20.54%)	258	3.61 ± 1.07
Q4: The law supports individuals’ rights to refuse or consent to treatment, where possible.	10 (3.88%)	23 (8.91%)	59 (22.87%)	121 (46.90%)	45 (17.44%)	258	3.65 ± 1.00
Q5: The law supports the fair and independent review of an individual’s detention.	13 (5.04%)	14 (5.43%)	81 (31.40%)	114 (44.19%)	36 (13.95%)	258	3.57 ± 0.97
Q6 *: The law is difficult to implement in clinical practice.	21 (8.14%)	90 (34.88%)	72 (27.91%)	63 (24.42%)	12 (4.65%)	258	3.17 ± 1.04
Q7 *: Information about the law is not readily available.	24 (9.30%)	69 (26.74%)	63 (24.42%)	83 (32.17%)	19 (7.36%)	258	2.98 ± 1.12
Q8: The transfer to the inpatient ward works well under the law.	18 (6.98%)	35 (13.57%)	76 (29.46%)	111 (43.02%)	18 (6.98%)	258	3.29 ± 1.02
Q9: Individuals admitted without their consent receive the least restrictive and most effective care available under the circumstances.	17 (6.59%)	56 (21.71%)	83 (32.17%)	86 (33.33%)	16 (6.20%)	258	3.11 ± 1.03

Note: Data are presented as frequency (%) unless otherwise mentioned. SD: standard deviation. * These items were reverse-scored to calculate agreement scores.

**Table 3:** Stakeholders’ opinions about the Saudi Mental Health Care Law across different domains.

		Specialty				
	Domains	Nursing (n = 36)	Psychiatry (n = 148)	Psychology (n = 70)	Social Work (n = 4)	*p*-Value
Q1	Treatment efficacy	4 (3.25–4)	4 (3–4.75)	4 (3–4)	3.5 (3–4)	0.031 *
Q2	Admission criteria	4 (3–4)	4 (3–4)	4 (3–4)	3.5 (1.5–4)	0.721
Q3	Care benefits	4 (2–4)	4 (3–5)	3 (2–4)	4 (3.25–4.75)	<0.001 **
Q4	Consent treatment	4 (2.25–4)	4 (3–4)	4 (3–4)	3 (3–3.75)	0.127
Q5	Detention review	4 (3–4)	4 (3–4)	4 (3–4)	4 (3.25–4)	0.175
Q6	Implementation of the law	3.5 (2–4)	3 (2–4)	3 (2–4)	3 (2.25–3.75)	0.949
Q7	Information about the law	3 (2–4)	3 (2–4)	3 (2–4)	2.5 (2–3.75)	0.274
Q8	Transfer to hospital	4 (2.25–4)	3 (3–4)	3 (2–4)	3.5 (3–4.75)	0.428
Q9	Reciprocity principle	3.5 (3–4)	3 (2–4)	3 (3–4)	2 (1.25–2.75)	0.055

Note: Data are presented as median (interquartile range), and statistical significance is set at *p*-value < 0.05. * Psychiatrists scored higher than psychologists. ** Psychiatrists scored higher than nurses and psychologists.

**Table 4:** Ordinal logistic regression models for factors associated with different mental healthcare law domains.

	Q1	Q2	Q3	Q4	Q5	Q6	Q7	Q8	Q9
Age (years)									
<30	Ref								
30–40	0.7	1.17	1.19	0.7	1.47	0.93	0.74	0.74	1.19
41–50	0.5	1.8	1.53	1.37	1.56	0.96	1.39	0.51	0.51
>50	0.3	1.3	1.35	0.84	1.51	1.01	1.07	0.53	0.52
Sex									
Male	Ref								
Female	0.87	1.2	0.92	1.3	0.87	0.95	1.43	1	0.76
Nationality									
Non-Saudi	Ref								
Saudi	0.71	0.75	0.93	1.66	1.37	1.22	1.13	0.57	0.33 *
Current region of practice									
Central	Ref								
Eastern	1.39	0.93	1.62	1.49	0.83	0.66	0.76	0.78	0.91
Northern	2.08	3.3 *	3.03	0.65	5.33 **	0.39	1.38	3.82 *	4.16
Southern	0.65	0.84	0.56	1.22	0.71	0.38	1.2	0.78	0.54
Western	1.25	1.34	1.42	1.03	1.2	1.35	1.67	1.84	0.67
Level of the hospital facility									
Primary care	Ref								
Secondary hospital	0.59	0.71	0.99	0.62	1	1.07	1.11	0.78	0.63
Tertiary hospital	0.88	0.65	1.05	0.57	1.03	1.46	1.59	0.99	0.89
Facility service type									
Governmental	Ref								
Private sector	0.99	1.07	1.13	0.97	2	0.86	0.93	1.41	0.91
Years of experience									
<2 years	Ref								
>2–5 years	1.26	1.11	0.89	1.31	0.96	0.65	0.58	1.37	0.96
>5–10 years	1.15	1.11	0.7	0.98	0.68	0.76	0.66	2.19	0.59
>10 years	1.55	0.76	0.96	0.89	0.94	0.88	0.89	1.19	1.18
Specialty									
Nursing	Ref								
Psychiatry	1.29	1.23	2.47 *	1.49	1.75	0.69	1.37	0.79	0.92
Psychology	0.62	0.82	0.77	1.15	0.66	1.01	0.88	0.58	1.45
Social work	0.57	0.58	3.37	0.55	1.95	0.47	0.57	2.68	0.21
Opinion on confidentiality									
The law has no negative effect on patients’ confidentiality.	Ref								
The law has a negative effect on patients’ confidentiality.	0.2 ***	0.44 *	0.59	0.35 *	0.35 *	0.24 **	0.59	0.43 *	0.6
I’m not sure.	0.37 ***	0.42 **	0.63	0.47 **	0.36 ***	0.42 **	0.49 **	0.65	1.19
Law implemented in participants’ facility									
I’m not sure.	Ref								
Neither implemented nor followed	0.24 **	0.33 *	0.38	0.32 *	0.27 *	1.04	0.54	0.95	0.56
Followed but not implemented officially	1.05	0.88	1.35	0.89	0.76	0.79	0.48	1.1	1.46
Officially implemented	1.57	1.29	1.3	1.07	1	1.21	0.96	1.89	2.09 *
Reading the law									
No	Ref								
No, but planning to	1.73	1.44	1.05	1.1	1.25	0.95	0.85	1.01	1.43
Yes	1.88	1.36	0.86	1.59	1.32	0.77	1.77	0.84	1.35
Attendance at lectures and workshops									
No	Ref								
No, but planning to	1.53	0.91	0.59	0.52 *	0.66	1.41	1.58	1.41	0.74
Yes	1.88 *	1.36	0.94	1.13	1.34	1.38	1.52	1.72	1.16

Note: The results are expressed as odds ratios, where *, **, and *** indicate *p*-value < 0.05, <0.01, and <0.001, respectively.

**Table 5:** Linear regressions model for factors associated with the total score recorded on the Mental Health Legislation Attitudes Scale.

	Univariate			Multivariable		
	Coefficient	95%CI	*p*-Value	Coefficient	95%CI	*p*-Value
Age (years)						
<30	Ref			Ref		
30–40	−0.12	−1.57 to 1.33	0.874	−0.3	−2.16 to 1.56	0.754
41–50	0.39	−1.83 to 2.6	0.731	0.02	−2.81 to 2.85	0.989
>50	−0.37	−2.94 to 2.2	0.778	−1.13	−4.53 to 2.27	0.512
Sex						
Male	Ref			Ref		
Female	−0.35	−1.65 to 0.96	0.602	0.18	−1.21 to 1.56	0.802
Nationality						
Non-Saudi	Ref			Ref		
Saudi	−1.75	−3.69 to 0.18	0.075	−0.71	−2.92 to 1.49	0.525
Current region of practice						
Central	Ref			Ref		
Eastern	0.03	−1.52 to 1.58	0.97	−0.08	−1.7 to 1.54	0.921
Northern	1.31	−1.62 to 4.24	0.38	2.66	−0.22 to 5.53	0.07
Southern	−1.7	−4.85 to 1.45	0.288	−1.84	−4.94 to 1.27	0.245
Western	0.6	−1.57 to 2.77	0.588	0.91	−1.18 to 3.01	0.392
Level of the hospital facility						
Primary care	Ref			Ref		
Secondary hospital	−0.28	−2.15 to 1.58	0.765	−0.73	−2.6 to 1.14	0.444
Tertiary hospital	0.73	−0.88 to 2.34	0.374	−0.13	−1.85 to 1.59	0.88
Facility service type						
Governmental	Ref			Ref		
Private sector	−0.69	−2.41 to 1.04	0.435	0.79	−1.32 to 2.9	0.463
Years of experience						
<2 years	Ref			Ref		
>2–5 years	−0.02	−1.81 to 1.76	0.978	0.09	−1.8 to 1.97	0.926
>5–10 years	−0.14	−2.13 to 1.85	0.889	−0.28	−2.77 to 2.22	0.828
>10 years	0.21	−1.63 to 2.04	0.823	0.07	−2.64 to 2.78	0.959
Specialty						
Nursing	Ref			Ref		
Psychiatry	1.28	−0.65 to 3.2	0.194	1.18	−0.88 to 3.23	0.26
Psychology	−0.33	−2.45 to 1.8	0.762	−0.76	−3.32 to 1.8	0.558
Social work	−1.06	−6.52 to 4.41	0.704	−0.66	−6.12 to 4.8	0.812
Opinion on confidentiality						
Law has no negative effect on patients’ confidentiality	Ref			Ref		
Law has a negative effect on patients’ confidentiality	−4.37	−6.32 to −2.43	<0.001 ***	−4.26	−6.28 to −2.24	<0.001 ***
I am not sure	−3	−4.36 to −1.64	<0.001 ***	−2.67	−4.1 to −1.24	<0.001 ***
Law implemented in participants’ facility						
I am not sure	Ref			Ref		
Neither implemented nor followed	−1.98	−4.54 to 0.58	0.128	−3.71	−6.35 to −1.06	0.006 **
Followed but not implemented officially	0.5	−1.47 to 2.47	0.618	−0.1	−2.12 to 1.93	0.926
Officially implemented	2.25	0.62 to 3.88	0.007 **	1.21	−0.51 to 2.93	0.167
Reading the law						
No	Ref			Ref		
No, but planning to	1.19	−0.88 to 3.27	0.258	0.83	−1.24 to 2.89	0.431
Yes	1.99	0.05 to 3.93	0.044 *	0.87	−1.12 to 2.85	0.39
Attendance at lectures and workshops						
No	Ref			Ref		
No, but planning to	0.34	−1.41 to 2.09	0.704	0.04	−1.7 to 1.77	0.967
Yes	1.81	0.27 to 3.35	0.021 *	1.25	−0.32 to 2.83	0.119

Note: CI: Confidence interval, *, **, and *** indicate *p*-value < 0.05, <0.01, and <0.001, respectively.

## Data Availability

The data that support the findings of this study are available from the corresponding author upon reasonable request.

## References

[1] Sidhu N., Srinivasraghavan J. (2016). Ethics and medical practice: Why psychiatry is unique. Indian J. Psychiatry.

[2] Simon R.I. (2003). The law and psychiatry. Focus.

[3] Funk M.K., Drew N.J. (2015). Mental health legislation. East. Mediterr. Health J..

[4] Minkowitz T. (2007). The United Nations Convention on the Rights of Persons with Disabilities and the right to be free from nonconsensual psychiatric interventions. Syracuse J. Int’l L. Com..

[5] Wilson K. (2021). Mental Health Law: Abolish or Reform?.

[6] Georgieva I., Whittington R., Lauvrud C., Steinert T., Wikman S., Lepping P., Duxbury J., Snorrason J., Mihai A., Berring L.L. (2019). International variations in mental-health law regulating involuntary commitment of psychiatric patients as measured by the Mental Health Legislation Attitudes Scale. Med. Sci. Law.

[7] Wasserman D., Apter G., Baeken C., Bailey S., Balazs J., Bec C., Bienkowski P., Bobes J., Ortiz M.F.B., Brunn H. (2020). Compulsory admissions of patients with mental disorders: State of the art on ethical and legislative aspects in 40 European countries. Eur. Psychiatry.

[8] Saya A., Brugnoli C., Piazzi G., Liberato D., Di Ciaccia G., Niolu C., Siracusano A. (2019). Criteria, procedures, and future prospects of involuntary treatment in psychiatry around the world: A narrative review. Front. Psychiatry.

[9] Greenland C. (1969). Appealing against commitment to mental hospitals in the United Kingdom, Canada, and the United States: An international review. Am J Psychiatry..

[10] Georgieva I., Bainbridge E., McGuinness D., Keys M., Brosnan L., Felzmann H., Maguire J., Murphy K., Higgins A., McDonald C. (2017). Opinions of key stakeholders concerning involuntary admission of patients under the Mental Health Act 2001. Ir. J. Psychol. Med..

[11] Al Habeeb A., Qureshi N. (2010). Mental and Social Health Atlas I in Saudi Arabia: 2007–08. East. Mediterr. Health J..

[12] Alqahtani A.H. (2020). Psychiatric admissions. Int. J. Risk Recov..

[13] Carlisle J. (2018). Mental health law in Saudi Arabia. BJPsych Int..

[14] Qureshi N.A., Al-Habeeb A.A., Koenig H.G. (2013). Mental health system in Saudi Arabia: An overview. Neuropsychiatr. Dis. Treat..

[15] World Health Organization (2005). WHO Resource Book on Mental Health, Human Rights and Legislation: Stop Exclusion, Dare to Care.

[16] World Health Organization (2013). Mental Health Action Plan 2013–2020. https://iris.who.int/handle/10665/89966.

[17] World Health Organization (2019). The WHO Special Initiative for Mental Health (2019–2023) Universal Health Coverage for Mental Health. https://iris.who.int/handle/10665/310981.

[18] State of Queensland (Queensland Health) (2016). About the Mental Health Act. https://www.health.qld.gov.au/clinical-practice/guidelines-procedures/clinical-staff/mental-health/act/about.

[19] State of Queensland (Queensland Health) (2016). A Guide to the Mental Health Act. https://www.health.qld.gov.au/__data/assets/pdf_file/0031/444856/guide-to-mha.pdf.

[20] Saudi Ministry of Health (2014). Mental Health Care Law. https://www.moh.gov.sa/en/Ministry/Rules/Documents/Menatl-Health-Care-Law.pdf.

[21] Saudi Systems Group (2014). Mental Healthcare System. https://laws.boe.gov.sa/BoeLaws/Laws/LawDetails/107f22b5-81a2-47ee-84bc-a9a700f2907a/1.

[22] Saudi Ministry of Health (2022). Statistical Yearbook. https://www.moh.gov.sa/en/Ministry/Statistics/book/Pages/default.aspx.

[23] Patricio A.L., Verdeprado R.H. (2020). Awareness on the mental health law of registered social workers in Negros Occidental. Philipp. Soc. Sci. J..

[24] Lepping P., Steinert T., Gebhardt R.P., Röttgers H.R. (2004). Attitudes of mental health professionals and lay-people towards involuntary admission and treatment in England and Germany—A questionnaire analysis. Eur. Psychiatry.

[25] Johnstone L., Boyle M. (2018). The power threat meaning framework: An alternative nondiagnostic conceptual system. J. Humanist. Psychol..

[26] McHale J.V. (2009). Patient confidentiality and mental health: Part 1. Br. J. Nurs..

[27] Fiorillo A., De Rosa C., Del Vecchio V., Jurjanz L., Schnall K., Onchev G., Alexiev S., Raboch J., Kalisova L., Mastrogianni A. (2011). How to improve clinical practice on involuntary hospital admissions of psychiatric patients: Suggestions from the EUNOMIA study. Eur. Psychiatry.

[28] Royal College of Psychiatrists Registration: Approved Clinician Induction. https://www.rcpsych.ac.uk/events/conferences/s12ACtraining/registration-approved-clinician.

[29] Zigmond T., Brindle N. (2022). A Clinician’s Brief Guide to the Mental Health Act.

[30] Rigby D., McAlpine L. (2019). Section 12 approval: Fit for purpose?. BJPsych Bull..

